# Group A streptococcal genotypes from throat and skin isolates in the United Arab Emirates

**DOI:** 10.1186/1756-0500-3-94

**Published:** 2010-04-06

**Authors:** Mubarak S Alfaresi

**Affiliations:** 1 Department of pathology & Laboratory medicine, Zayed Military Hospital, PO BOX 3740, Abudhabi, UAE

## Abstract

**Background:**

The bacterium *Streptococcus pyogenes* causes a variety of human diseases that range from relatively mild skin infections to severe invasive diseases, such as acute rheumatic fever, glomerulonephritis, puerperal sepsis, necrotizing fasciitis, meningitis, and streptococcal toxic shock syndrome. Accurate identification and typing of group A hemolytic streptococci (GAS) is essential for epidemiological and pathogenetic studies of streptococcal diseases. For this reason, The genetic diversity of group A streptococcal (GAS) isolates from subjects in the United Arab Emirates with streptococcal disease was studied using *emm *gene sequence analysis. The emm typing system which is based on sequence analysis of PCR products of the N-terminal hypervariable region of the M protein gene, concurs with M serotyping almost 1:1.

**Findings:**

A total of 38 GAS isolates were analyzed, including 35 isolates from throat and 3 from skin. Among the 38 isolates, a total of 25 different *emm/st *types were detected: 20 isolates (53%) belonged to 16 validated standard reference *emm *types and 18 isolates (47%) belonged to 9 recognized sequence types.

**Conclusions:**

This is the first *emm *typing study in the United Arab Emirates to demonstrate the heterogeneity of the GAS population.

## Findings

### Introduction

The bacterium *Streptococcus pyogenes* causes a variety of human diseases that range from relatively mild skin infections to severe invasive diseases, such as acute rheumatic fever, glomerulonephritis, puerperal sepsis, necrotizing fasciitis, meningitis, and streptococcal toxic shock syndrome [1-3].

Accurate identification and typing of group A hemolytic streptococci (GAS) is essential for epidemiological and pathogenetic studies of streptococcal diseases. Rebecca Lancefield developed a serotyping system based on antigenic variation of the cell surface M protein that has been in use since 1928. Although additional serotyping systems, i.e. T and OF typing, were developed as valuable and practical substitutes, the M typing system has been considered the gold standard method. However, a limited supply of antisera and the high nontypeability rate among isolates, in particular among isolates from the tropics, present a challenge to using the M typing system. In recent years, several molecular typing systems have been developed as alternatives to M typing [4-7].

Numerous typing schemes have been used to characterize and measure genetic diversity among *S. pyogenes* isolates. Perhaps the most common tool used today is emm typing. The emm typing system [4,6,7], which is based on sequence analysis of PCR products of the N-terminal hypervariable region of the M protein gene, concurs with M serotyping almost 1:1. In addition to its simplicity, emm typing has allowed the detection of several previously unknown GAS types from different geographic regions [8-11]. Knowledge of the dynamics of emm types in a given region may shed light on the pathogenesis of GAS infections and is critical for selecting appropriate vaccine candidates.

It has been noted that there are differences in the distribution of GAS M-types in tropical versus temperate regions, since antisera produced against strains from Europe and the United States do not recognize large proportions of isolates from the tropics. This implies that a multivalent vaccine targeting the most common emm types in another part of the world may be ineffective in the tropics. In this study, we surveyed the genetic diversity of GAS in the UAE using emm gene sequence analysis. We wished to better understand the epidemiology of this important pathogen in a country where complications resulting from streptococcal infections are still common. This study is the first to report emm typing results in the UAE. The findings demonstrate the divergent features of GAS in this region.

## Materials and methods

### Ethical Approval

This study was reviewed and approved by Zayed Military Hospital. Patients were managed under standard approved hospital procedures.

### Bacterial isolates

The GAS isolates in the present study were obtained in the UAE from January 2009 to December 2009 from patients with signs of streptococcal disease. Patients are from different ethnic groups (multinational). A total of 38 GAS isolates were included: 35 isolates from throat and 3 from skin. The isolates were associated with cases of acute tonsillitis (n = 35) and impetigo (n = 3). All GAS isolates were subjected to biochemical classification using the VITEK 2 system (bioMérieux) and SLIDEX STREPTO Plus latex agglutination kit (bioMérieux) to further verify that they belonged to *Streptococcus pyogenes* (group A). This collection of isolates was tested previously for antibiotic resistance to commonly used antibiotics using the disk diffusion method [12].

### emm typing

emm typing was performed according to the protocol described by the Centers for Disease Control and Prevention (CDC; http://www.cdc.gov/ncidod/biotech/strep/protocols.html). DNA templates were prepared using the QIAGEN bacterial extraction kit. Briefly, for each isolate, about half a loop of bacterial cells was picked from overnight growth on blood agar plates and suspended in 100 μl of distilled water. This suspension was then used for bacterial extraction according to the instructions for the kit. The eluted DNA was used immediately for PCR or else was stored at -20°C until use. Primers 1 and 2, as specified by the CDC protocol, were used for amplifying the N-terminal region of the emm gene: primer 1, 5-TAT TCG CTT AGA AAA TTA A-3 and primer 2, 5-GCA AGT TCT TCA GCT TGT TT-3. AmpliTaq Gold (Applied Biosystems) PCR reagents were used according to the manufacturer's instructions. Amplification was performed on a PCR thermal cycler (GeneAmp*, PCR System 9700; Applied Biosystems) using cycle parameters specified by the CDC. Amplification products were purified using the JetQuick spin column technique (Germond) according to the manufacturer's instructions. PCR products were analyzed on an agarose gel to ensure the quality of the DNA and to estimate the concentration for sequencing.

Primer emmseq2, 5-TAT TCG CTT AGA AAA TTA AAA ACA GG-3, or primer 1 was used for sequencing the PCR products using the BioDye Terminator Cycle Sequencing Ready Reaction kit (version 2.0; BioSystems). Primer 1 was used when emmseq2 sequencing did not give good results. The sequencing reaction mixtures were prepared according to the kit instructions using the following parameters: 96°C for 1 min; 25 cycles of 96°C for 10 s; 50°C for 5 s; and 60°C for 4 min. The holding temperature was 4°C. The sequencing products were purified using ethanol sodium acetate precipitation and subjected to automated sequence analysis on an ABI Prism 310 Genetic Analyzer. The Geneious genetic software program (version 4.7.5) was used to edit the sequences. The 5' end of the sequences was compared to sequences in the CDC database http://www.cdc.gov/ncidod/biotech/strep/strepblast.htm. Sequences that match sequences in this database were checked for similarity with the published sequences in the GenBank database http://www.ncbi.nlm.nih.gov/BLAST/.

### GenBank accession numbers

Sequences from the emm gene that were acquired during this study were deposited in the GenBank under numbers HM015360-HM015397.

### Tests for significance

The level of significance was determined by calculating the P value using the chi-square test.

## Results

### Emm type distribution

Table 1 shows the *emm/st *type, site of isolation, and disease association of the 38 GAS isolates in this study. "*emm*" refers to the type-specific sequence of one of the standard reference GAS strains, and "st" stands for sequence types discovered later, including novel sequence types in the present study that await validation by the international committee http://www.cdc.gov/ncidod/biotech/strep/strepblast.htm[13]. A total of 25 different emm/st types were detected among the 38 GAS isolates. Of these 38 isolates, 20 (35%) belonged to 16 validated standard reference emm types [13,14], and 18 isolates (47%) belonged to 9 recognized sequence types http://www.cdc.gov/ncidod/biotech/strep/strepblast.htm.

**Table 1:** M protein gene (*emm*) type distribution in relation to disease and isolation site.

			Site of isolation		Disease status	
			
*emm *type	Total no. of isolates	%	Skin	Throat	Impetigo	Tonsillitis
*emm89*	3	7.9		3		3
*st4695.0*	2	5.3	2		2	
*emm83.8*	1	2.6		1		1
*emm78.3*	2	5.3		2		2
*st3211.0*	4	10.5		4		4
*st75.0*	2	5.3		2		2
*112.2*	1	2.6		1		1
*stIL103.1*	1	2.6		1		1
*emm1*	1	2.6		1		1
*stIL62.0*	2	7.9		2		2
*emm75.2*	1	2.6		1		1
*emm100.5*	1	2.6		1		1
*emm4.9*	1	2.6		1		1
*emm4.4*	1	2.6		1		1
*emm120*	1	2.6		1		1
*stC6979.1*	1	2.6		1		1
*st0721.0*	3	7.9		3		3
*emm75*	2	5.3		2		2
*stG97.0*	1	2.6		1		1
*emm71.3*	1	2.6		1		1
*emm22.1*	1	2.6	1		1	
*emm22*	1	2.6		1		1
*emm83.1*	1	2.6		1		1
*st9505.0*	1	2.6		1		1
*emm87*	1	2.6		1		1

Total	38	100	3	35	3	35

**Figure 1:**
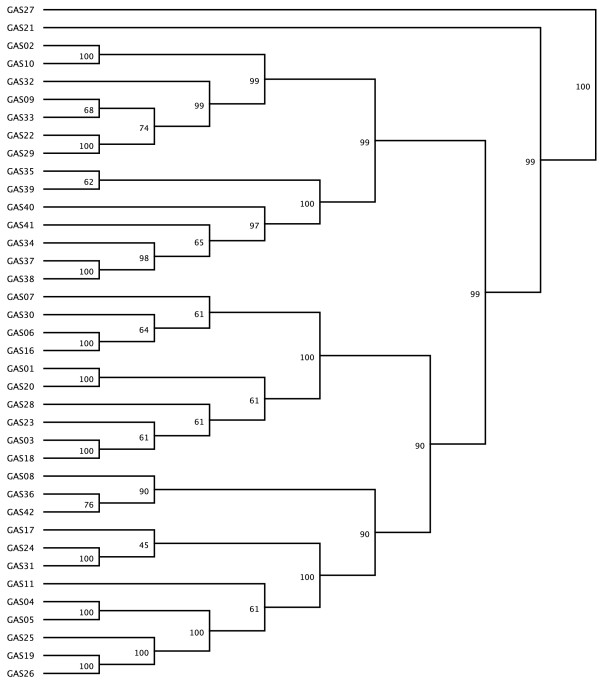
**Dendrogram for the 38 GAS emm gene sequences according the the isolate number**. The dendrogram was constructed with Geneious V4.8 sofware, by using the UPGMA algorithm.

To compare the clonal relationships between the isolates, a dendrogram was generated with the UPGMA algorithm (Fig. 1).

In contrast to most studies in which a large proportion of the GAS isolates belong to a few *emm *types, there were no dominant emm types in the present study. Eight different emm types (listed in descending order: st3211.0, st0721.0, emm89, emm 75, st4695.0, st75.0, stIL62.0, and emm78.3) made up 55.4% of the isolates (Table 1). The most common type, st3211.0, comprised only 10.5% of the total isolates (4 isolates). Interestingly, types emm12, emm18, emm3, emm4, which have remained among the most frequent emm types for decades in the Western world [15-17], were absent in this collection. Type emm89, which is frequent in Canada [17], was also frequent in this study.

### *emm *types of skin and throat isolates

The concept of distinct throat and skin emm types has been widely accepted. M protein serotypes, such as M types 1, 3, 4, 5, 6, 12, 14, 18, 19, and 24 of *S. pyogenes*, are associated with throat infections [18], while M serotypes such as 2, 49, 57, 59, 60, and 61 are associated with impetigo [19]. The number of isolates of each emm type in the present study is too low make statistical correlations regarding tissue specificity. However, it is worth noting that a number of emm types with ≥3 isolates, namely st3211.0, stIl62.0, st0721.0, and emm89, were isolated only from throat samples. One emm type with two isolates, st4695.0, was isolated only from the skin.

## Discussion

The findings in this study demonstrated that the GAS isolates from patients in the UAE are highly heterogeneous, with 25 distinct emm/st types detected among the 38 isolates that were collected over a 1-year period. The number of distinct emm/st types detected in this study, 25, is not much higher than the number reported in emm typing studies conducted in other countries that involved more isolates collected over a longer period of time[2,16,17,19,20]. In Japan, 29 emm/st types were detected among 906 clinical isolates collected from 1990 to 1999 [21]. In Mexico, 31 emm types were detected among 423 isolates collected from symptomatic pharyngeal specimens from 1991 to 2000 [20]. In a population-based study among the Australian aboriginal population, 31 distinct types were reported among 141 isolates collected from the skin and throats of asymptomatic cases over a 25-month period [8]. In a nationwide survey in Canada, 54 M serotypes were reported among 4,760 GAS isolates submitted to the national center for streptococci from 1993 to 1999 [17]. In Spain, only 30 different emm types were detected among 614 pharyngeal isolates collected from eight different hospitals over a 4-year period[22]. The reason for the relatively higher heterogeneity observed among the UAE isolates can be attributed to the heterogeneity of the UAE population due to many international immigrants.

Since the introduction of the GAS sequence typing method based on the heterogeneity of the 5' end of the emm gene [4,6], several previously undocumented emm types have been detected. The number of distinct emm/st types has more than doubled in the last few years, and currently the CDC database contains over 225 distinct emm/st types. In the present study, there were no new emm types among the isolates. This might due to the small number of isolates. The detection rate of undocumented emm types has varied from country to country. In general, more new types have been reported in Malaysia [9], Thailand [10], Brazil [11], the United States [23], and Australia [8] compared than in European countries.

The highly diverse nature of the GAS population in the UAE calls into question the concept of using a multivalent vaccine composed of the N-terminal regions of the most common emm types. A multivalent vaccine composed of 26 M protein N-terminal regions was anticipated to protect against ca. 90% of the invasive GAS infections in the United States [24]. Designing an effective multivalent vaccine based on the N-terminal regions of M protein for countries such as the UAE, where the GAS population is highly heterogeneous and the dynamics of streptococcal infections are poorly understood, presents a challenge. Based on the observations in the present study, a vaccine with at least 25 different emm type-specific epitopes would be required to cover ca. 80% of the GAS infections in the UAE.

The aim of this study was to survey the genetic diversity of GAS isolates from the UAE using emm gene sequence analysis. Our GAS collection contained isolates from the skin and throats of symptomatic individuals, and the findings demonstrated the highly heterogeneous nature of the GAS population in the UAE during the study period. This study is the first of its kind to be conducted in samples from the UAE, and it underscores the unique nature of GAS epidemiology in this region. To better understand the dynamics of GAS epidemiology in the tropics, further surveillance in the region is needed. This may also help elucidate the pathogenesis of this bacterium, as well as help design an appropriate vaccine.

## Competing interests

The author declares that he has no competing interests.

## Authors' contributions

All the work was conducted by the author himself.
